# Mandibular Block Graft for Localised Ridge Augmentation Followed by Delayed Implant Placement: A Case Report

**DOI:** 10.7759/cureus.34881

**Published:** 2023-02-11

**Authors:** Pallavi Ghodpage, Amit Suroshe

**Affiliations:** 1 Department of Periodontology, Seema Dental College, Rishikesh, IND; 2 Department of Periodontology, Suroshe Dental Clinic, Mumbai, IND

**Keywords:** esthetics, chin graft, implants, ridge augmentation procedure, autogenous bone graft

## Abstract

The presence of adequate ridge width is an important prerequisite for implant placement. Reconstruction of the alveolar ridge can be done through various bone augmentation procedures. Autogenous bone grafts are being used for ridge augmentation for a long time and are still considered the gold standard for jaw reconstruction. Intraoral autogenous bone grafts from sites such as mandibular symphysis and ramus offer various advantages over the extraoral sites.

This case report describes the use of an autogenous block graft from mandibular symphysis for a ridge augmentation procedure in the maxillary anterior region followed by delayed implant placement. Six months postoperative clinical and radiographic examination revealed significant increase width of the ridge and appropriate implant placement with satisfactory esthetic and functional results.

## Introduction

Dental implants have evolved as a widely accepted treatment modality for restoring missing teeth. The appropriately osseointegrated implant serves as an excellent base on which implant-supported prosthesis can be delivered. The presence of adequate bone volume is an integral requirement for implant therapy.

Extraction of tooth or tooth loss is responsible for the resorption of the alveolar ridge. The width and height of the alveolar ridge rapidly decrease in the first year. 50 percent loss of width occurs in the first year, two third of which occurs in the initial 3 months. This leads to the formation of a defect in the bone. These defects may be a result of atrophy, dental trauma, accidents, pathologic resorption (inflammation, cyst formation), periodontal disease, and previous surgeries. Missing teeth with bone loss are associated with compromised mastication, swallowing and speech function, and psychological conditions, thus leading to functional and esthetic impairments that reduce patients' quality of life.

Implants are one of the reliable treatment modalities to replace missing teeth, but the presence of inadequate bone volume renders it difficult for a practitioner to place an implant at such sites. Bone reconstruction techniques have greatly improved to enhance the esthetic and functional results. Reconstruction of atrophic alveolar ridges still remains difficult in oral implantology. Intramembtranous autogenous grafts offer excellent osteogenic, osteoconductive, and osteoinductive properties. Hence they are still considered as the gold standard for alveolar ridge reconstruction, despite newer bone grafts and advanced bone grafting technologies being developed [1]. Autografts can be procured from either extraoral sites such as the iliac crest, or intraoral sites such as the ramus of the mandible, mandibular symphysis, and edentulous ridge.

An excellent corticocancellous bone graft can be obtained intraorally from symphysis or buccal shelf of the ramus. Approximately 65% cortical and 36% cancellous bone is found in an average symphysis graft which is enough to reconstruct a deficient ridge of 4-6 mm horizontal and 4mm vertical in dimension [2,3]. 

This is a case report of localized maxillary ridge augmentation using an autograft harvested from mandibular symphysis in conjugation with demineralized freeze-dried bone allograft (Tata memorial hospital, Mumbai).

## Case presentation

A 27-year-old male patient reported to the OPD of the Department of Periodontology, VSPM Dental College and Research Center, Nagpur, Maharashtra for replacement of his missing maxillary left central incisor. The patient lost his maxillary left central incisor 6 years ago in a road traffic accident and the area has been edentulous since then. The patient was systemically healthy, nonsmoker, reported no drug allergies, and was classified as ASA1 according to the American Society of Anaesthesiologists physical status classification.

**Figure 1:**
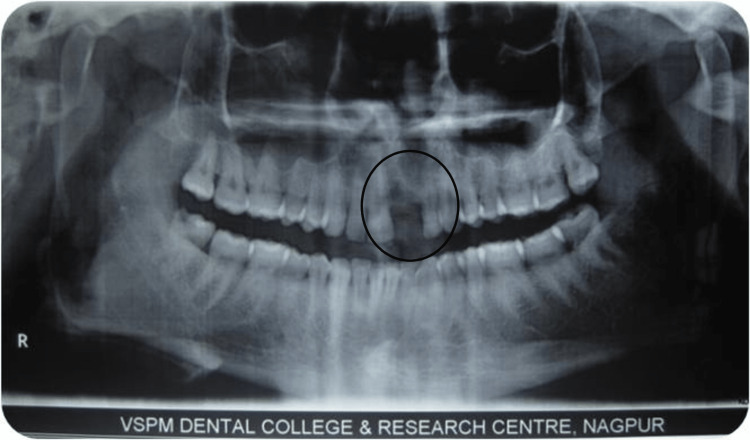
Preoperative OPG showing deficient ridge in the maxillary anterior region

A thorough clinical examination was carried out and an OPG was taken initially. Horizontal ridge resorption was seen at the maxillary left central incisor region (Figure 1).

After discussing all the possible treatment alternatives with the patient, the ridge augmentation procedure using an autogenous block graft harvested from the mandibular symphysis region and delayed implant placement was finalized. Signed informed consent was obtained from the patient.

The patient underwent thorough scaling and root planning, followed by oral hygiene instructions. After revaluation, the patient was recalled for surgery.

**Figure 2:**
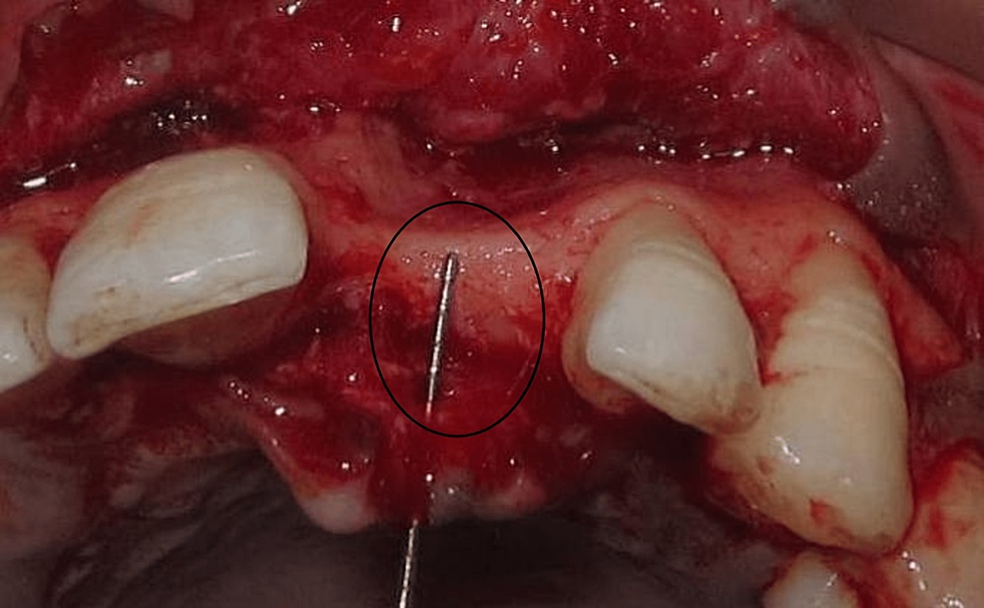
Ridge with of 3mm after reflection of full thickness flap

The surgical phase was performed on an outpatient basis under aseptic conditions. The patient was asked to rinse with 0.2% chlorhexidine for 1 min before surgery. The perioral skin disinfection was done with a 5% povidone-iodine solution. The surgical procedure was done under local anesthesia (2 % lignocaine with 1:80,000 adrenaline). Crestal incisions were given to gain access and intrasulcular incisions were given on buccal aspects of adjacent teeth along with releasing incisions. A full-thickness mucoperiosteal flap was reflected to expose the underlying alveolar bone. A bone width of 3mm was revealed which was inadequate for prosthetic rehabilitation (Figure 2).

**Figure 3:**
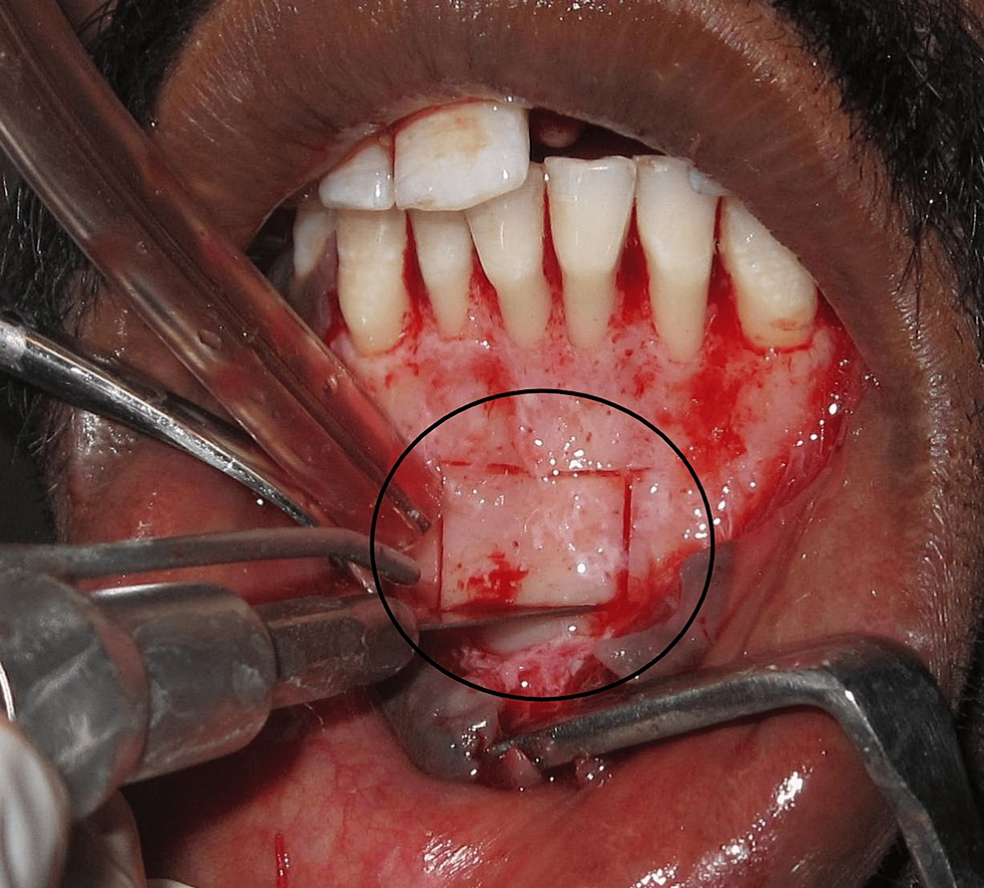
Donor site osteotomy

Fibrous tissue was removed, and decortication and perforations of marrow spaces were done with surgical burs to improve the vascularization and incorporation of the graft. The block graft was obtained from the mandibular symphysis region. Incisions were given at the sulcus which extended from the second bicuspid of the third quadrant to the second bicuspid of the fourth quadrant. The block graft was harvested using a trephine bur #5 and using straight and curved osteotomes (Figure 3).

**Figure 4:**
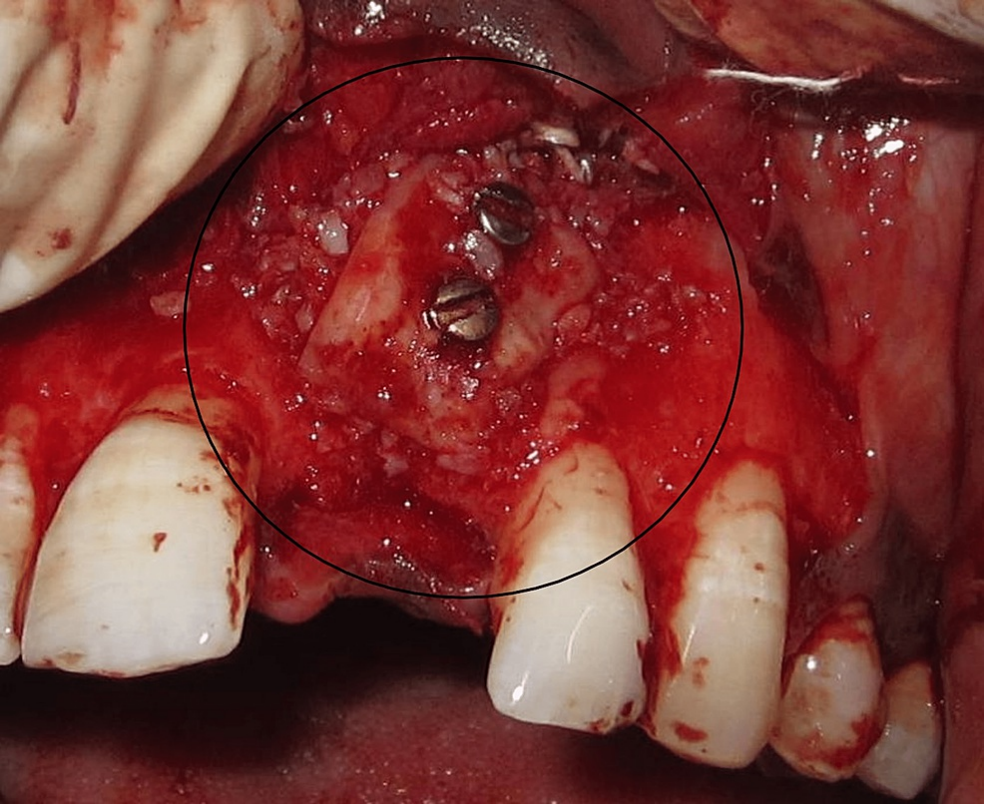
The block graft fixated to recipient site

The donor site was then packed with gel foam. Sutures were placed at the donor site for tension-free closure. The block graft was then transferred to the recipient site with fixation screws and the site was covered with a demineralized freeze-dried bone allograft (DFDBA) (Tata Memorial Hospital, Mumbai) (Figure 4).

**Figure 5:**
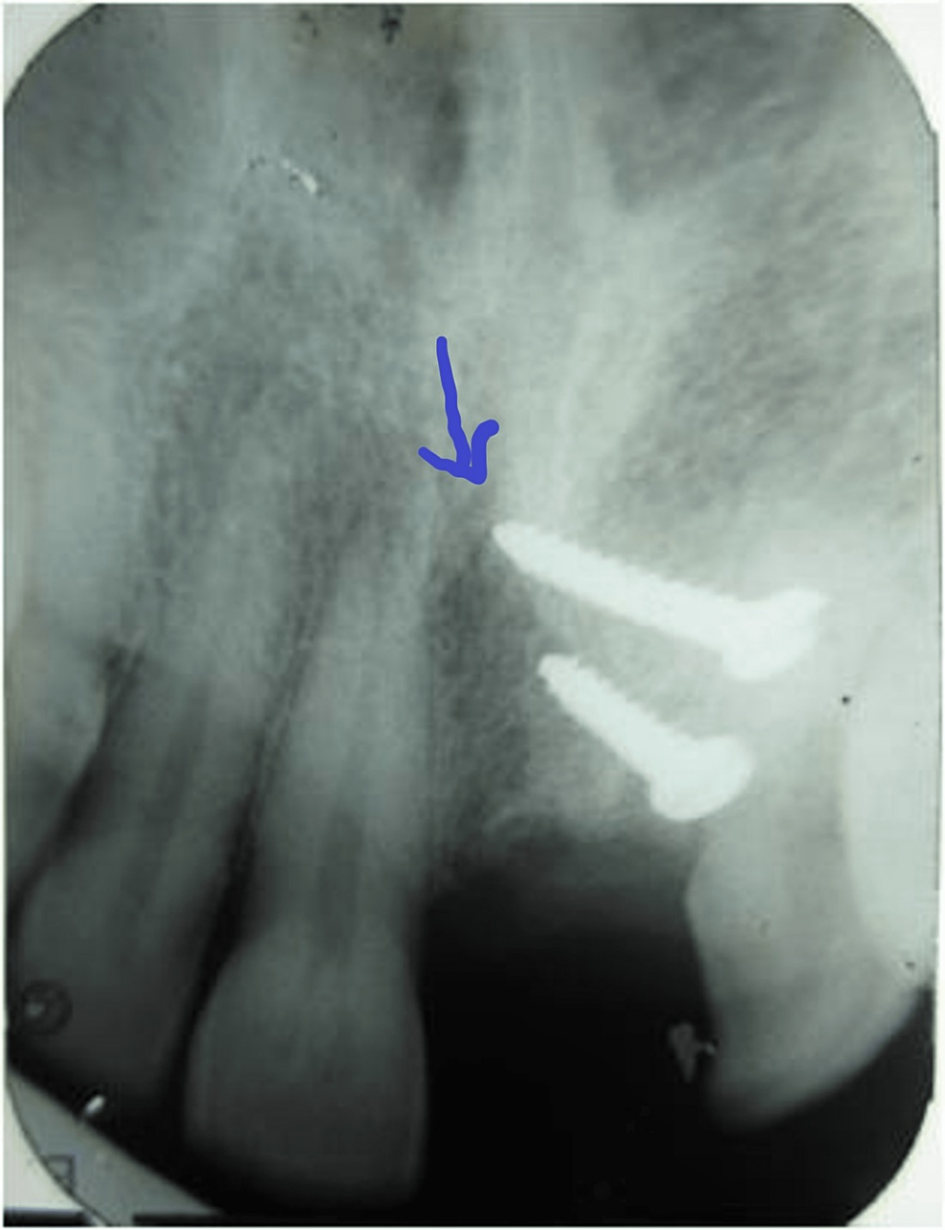
Intraoral periapical radiographs showing fixation screws

The recipient site was then sutured. Dexamethasone was given subcutaneously at the donor site. A post-operative intraoral periapical radiograph was taken (Figure 5).

**Figure 6:**
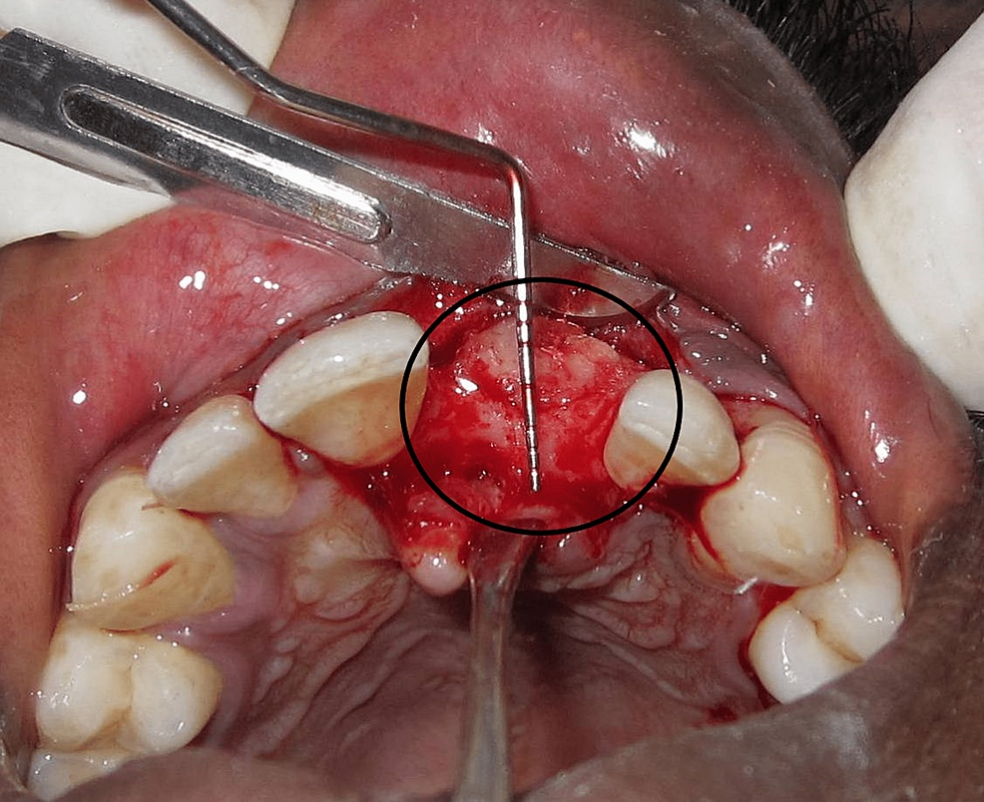
Ridge width of 7mm was obtained after the block graft placement

Antibiotics such as amoxicillin with clavulanic acid 625 mg BD for 5 days, Ibuprofen 400 mg TDS for 5 days, and 0.2% chlorhexidine mouth rinse twice daily for one week were prescribed postoperatively. The patient was recalled after 14 days and the sutures were removed. After 5 months of uneventful healing reentry, the procedure was performed. After the initial incision and reflection of the underlying bone, a ridge width of 7mm was obtained (Figure 6).

**Figure 7:**
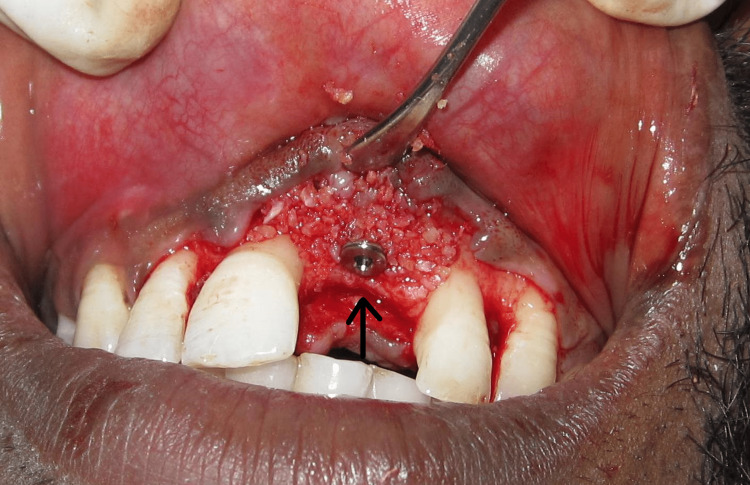
Implant placement

The fixation screws were removed. An implant was placed in the area which was 5mm in diameter and 11.5 mm in length. The insertion torque of >35 Ncm was obtained (Figure 7).

**Figure 8:**
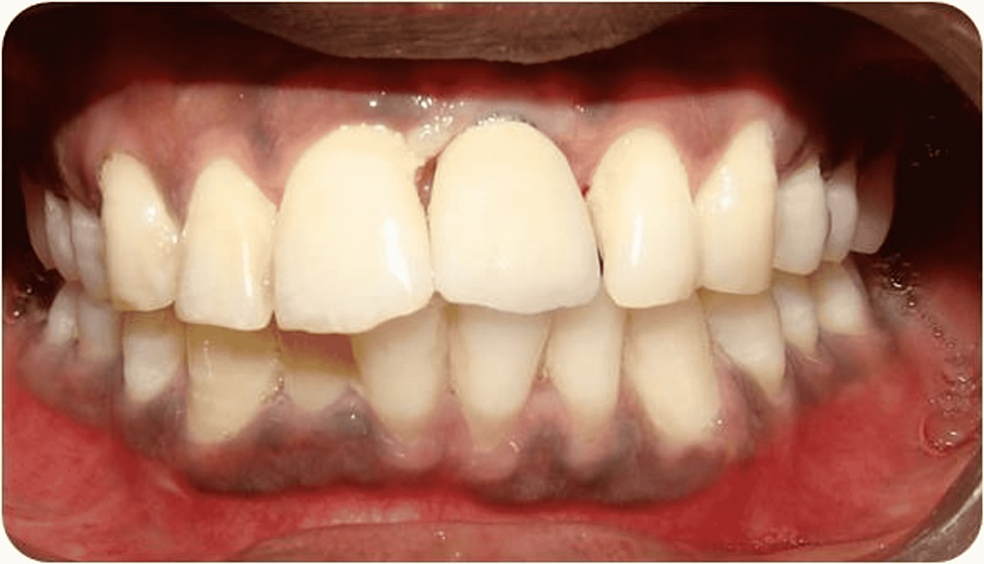
Final Prosthesis

After 5 months, the healing abutment was connected. After proper maturation of soft tissue, impressions were taken and a temporary prosthesis was given. After 6 months, an implant-supported cement-retained metal-ceramic prosthesis was delivered (Figure 8).

## Discussion

Prosthetic rehabilitation with implant therapy requires adequate height and width of the alveolar ridge. Different techniques such as distraction osteogenesis, bone splitting, and GTR are employed for bone augmentation depending upon the size of the defect. In the present case, the patient was young and systemically healthy and desired implant therapy. Therefore, ridge augmentation using autogenous block graft from the mandibular symphysis region and delayed implant placement were planned.

Autogenous bone grafting is considered a gold standard for bone transplantation. The major advantages are osteogenic potential, properties such as osteoinduction and osteoconduction, and no risk of graft rejection or adverse immunological reaction. The mandibular symphysis is an excellent source for ridge augmentation to obtain a corticocancellous bone. The corticocancellous nature of bone harvested from the mandibular symphysis facilitates faster vascularization, helps in rapid integration, and has less potential resorption during healing [4]. It provides D-1 (>1250 HU) or D-2 (850-1250 HU) density bone for augmentation [2]. The surgical access to the symphysis is easy with no cutaneous scar formation and the procedure can be performed on an outpatient basis [5].

Sakkas et al (2017) evaluated the autologous bone grafting following implant placement for 3 years where they concluded that autologous bone grafts prove to be a reliable entity for ridge augmentation owing to their low comorbidity. The results demonstrated a 95.6% high graft success rate and a very low early implant failure (0.38%). With these results, it can be concluded that the autogenous bone graft can be considered a "Gold Standard" for dentoalveolar reconstruction [6].

Flap dehiscence with or without exposure of the graft is among the most common postoperative complications [7]. In the present case, no soft tissue dehiscence was observed after the graft placement. One more common complication after the mandibular graft harvest was temporary paresthesia for up to 6 months and altered lower incisor sensations. To avoid this, the Rule of 5’s - a safe surgical technique proposed by Misch in 1992 to harvest a bone block from symphysis preventing injury to neurovascular components of the mandibular symphysis region was followed [8].

The application of bone graft (which has a low turnover rate) to cover the autogenous graft may reduce the resorption rate of the bone block. It acts as a scaffold for bone regeneration and fills the space between the graft and recipient site [9,10]. In the present study, DFDBA graft was used, advantages of DFDBA graft being enhanced osteogenic potential as stated by Urist [11].

## Conclusions

The ridge augmentation procedure of a deficient ridge is indeed a challenging and technique-sensitive procedure. Various techniques and materials for augmentation have been used which have given favorable results. However, the use of autogenous bone grafts still remains the “Gold standard”. The results of the present case showed that autogenous graft procured from the mandibular symphysis and delayed implant placement may provide adequate functional and aesthetic outcomes. However, additional long-term evaluation may be needed. 
